# Decision-Support for Restorative Dentistry: Hybrid Optimization Enhances Detection on Panoramic Radiographs

**DOI:** 10.3390/healthcare13222904

**Published:** 2025-11-14

**Authors:** Gül Ateş, Fuat Türk, Elif Tuba Akçın, Müjgan Güngör

**Affiliations:** 1 Department of Prosthodontics, Faculty of Dentistry, Yıldırım Beyazit University, 06800 Ankara, Turkey; gulates@aybu.edu.tr; 2 Department of Computer Engineering, Faculty of Technology, Gazi University, 06560 Ankara, Turkey; 3 Department of Prosthodontics, Faculty of Dentistry, Lokman Hekim University, 06510 Ankara, Turkey; tuba.akcin@lokmanhekim.edu.tr; 4 Department of Oral and Maxillofacial Radiology, Faculty of Dentistry, Lokman Hekim University, 06510 Ankara, Turkey; mujgan.gungor@lokmanhekim.edu.tr

**Keywords:** artificial intelligence (AI), dental restorations, panoramic dental radiographs, hybrid optimization (HGWO-PSO), convolutional neural network (CNN)

## Abstract

**Highlights:**

**What are the main findings?**

- A hybrid HGWO-PSO + SVM pipeline achieved the best five-class performance on panoramic radiographs (Accuracy 73.15%, macro-F1 0.728), outperforming a baseline CNN and conventional ML models.
- A patient-level 80/20 split and 5-fold CV showed stable results despite class imbalance; most errors occurred between radiopaque restorations (crowns vs. bridges).

**What are the implications of the main findings?**

- Optimization-assisted feature selection can provide a more balanced and interpretable alternative to end-to-end DL on small, single-center datasets, supporting real-world deployment.
- The system is decision-supportive for restorative dentistry—helping standardize re-porting and triage—while larger, multi-center datasets and stronger DL baselines are needed for broader clinical adoption.

**Abstract:**

**Background/Objectives:** Artificial intelligence (AI) has been increasingly used to support radiological assessment in dentistry. We benchmarked machine learning (ML), deep learning (DL), and a hybrid optimization-assisted approach for the automatic five-class image-level classification of dental restorations (filling, implant, root canal treatment, fixed partial denture/bridge, crown) on panoramic radiographs. **Methods:** We analyzed 353 anonymized panoramic images comprising 2137 labeled restorations, acquired on the same device. Images were cropped and enhanced (histogram equalization and CLAHE), and texture features were extracted with GLCM. A three-stage pipeline was evaluated: (i) GLCM-based features classified by conventional ML and a baseline DL model; (ii) Hybrid Grey Wolf–Particle Swarm Optimization (HGWO-PSO) for feature selection followed by SVM; and (iii) a CNN trained end-to-end on raw images. Performance was assessed with an 80/20 per-patient split and 5-fold cross-validation on the training set. While each panoramic radiograph may contain multiple restorations, in this study we modeled the task as single-label, image-level classification (dominant restoration type) due to pipeline constraints; this choice is discussed as a limitation and motivates multi-label, localization-based approaches in future work. The CNN baseline was implemented in TensorFlow 2.12 (CUDA 11.8/cuDNN 8.9) and trained with Adam (learning rate 1 × 10^−4^), with a batch size 32 and up to 50 epochs with early stopping (patience 5); data augmentation included horizontal flips, ±10° rotations, and ±15% brightness variation. A post hoc power analysis (G*Power 3.1; α = 0.05, β = 0.2) confirmed sufficient sample size (*n* = 353, power > 0.84). **Results:** The HGWO-PSO + SVM configuration achieved the highest accuracy (73.15%), with macro-precision/recall/F1 = 0.728, outperforming the CNN (68.52% accuracy) and traditional ML models (SVM 67.89%; DT 59.09%; RF 58.33%; K-NN 53.70%). **Conclusions:** On this single-center dataset, the hybrid optimization-assisted classifier moderately improved detection performance over the baseline CNN and conventional ML. Given the dataset size and class imbalance, the proposed system should be interpreted as a decision-supportive tool to assist dentists rather than a stand-alone diagnostic system. Future work will target larger, multi-center datasets and stronger DL baselines to enhance generalizability and clinical utility.

## 1. Introduction

A hallmark of the twenty-first century is the widespread use of digital technologies in personal and professional contexts. Technology dominates many aspects of daily life, and concepts such as “digital transformation,” “digital workflows,” and “digital technologies” have become commonplace. The extensive adoption of mobile devices, tablets, and smartphones, combined with easy access to technology and the internet, has significantly influenced societies’ cultural and professional habits [1].

Artificial intelligence (AI) broadly refers to computer systems designed to perform tasks that normally require human intelligence. It uses algorithms and computational models to emulate cognitive processes such as perception, reasoning, and decision-making [2]. Machine learning (ML) and deep learning (DL) are two main subfields of AI [3]. ML enables systems to automatically learn from data and improve performance without explicit programming, whereas DL employs multilayer artificial neural networks (ANN) to learn complex and abstract patterns [4,5,6]. A specialized ANN variant, the convolutional neural network (CNN), is particularly effective for analyzing two- and three-dimensional image data [7,8]. DL models, especially CNNs, have demonstrated high accuracy in image classification and detection, though they generally require larger datasets and higher computational power [9].

In recent years, AI has been increasingly adopted in restorative dentistry for the detection and classification of crowns, bridges, fillings, implants, and root-canal treatments on panoramic radiographs. However, many previous studies focus on a single restoration type or rely solely on end-to-end DL approaches, which demand extensive annotated datasets. In smaller single-center datasets, texture-based information can remain highly informative; thus, hybrid frameworks combining handcrafted feature extraction with optimization-driven classifiers can balance accuracy and interpretability under data constraints. Inter-observer agreement on 50 randomly selected cases yielded Cohen’s κ = 0.91, indicating excellent reliability.

AI applications have expanded across medicine and dentistry, particularly in prosthetic dentistry, which aims to restore oral function, esthetics, and comfort by replacing missing teeth and tissues while preserving the health of remaining dentition. Accurate pre-prosthetic planning requires comprehensive evaluation of existing restorations such as crowns, bridges, and implants. AI-based algorithms capable of automatically detecting these restorations on panoramic radiographs can support clinicians in diagnosis and treatment planning. CNNs have already been used successfully for tasks such as anatomical landmark detection, caries identification, tooth classification, and segmentation, with several studies reporting accuracy rates above 90% [10,11,12,13,14]. Beyond performance gains, AI can improve diagnostic consistency, reduce inter-observer variability, and decrease clinicians’ workload. Furthermore, restoration identification contributes to electronic patient record management and can assist in forensic identification and post-disaster victim recognition [15].

To enhance computer-aided diagnosis in dentistry, there is a growing need for models that efficiently integrate visual and spatial information [16,17]. Panoramic imaging—a widely utilized modality that captures a comprehensive view of the maxillofacial region in a single exposure while minimizing patient discomfort and radiation dose—plays a key role in this context [18,19]. AI methods in dental radiology have been explored for early detection of oral cancers, identification of jawbone fractures, assessment of temporomandibular joint osteoarthritis, and caries detection [20]. By improving diagnostic accuracy and efficiency, AI technologies hold promise for better patient outcomes. Although multiple restorations can coexist within a single panoramic image, the present study formulates the task as single-label, image-level classification focusing on the dominant restoration type. This design simplifies analysis for a limited dataset and is further discussed in Section 4 as a methodological limitation. The proposed framework is positioned as a diagnostic decision-support tool intended to assist clinicians rather than replace expert judgment.

**Accordingly, this study addresses the following research questions:**

(1) How accurately do different ML and DL algorithms detect and classify dental restorations in panoramic radiographs?
(2) How does a hybrid optimization algorithm such as HGWO-PSO enhance classification accuracy compared with standalone models?
(3) Which AI method provides the best balance between detection speed and diagnostic reliability for clinical use? By systematically evaluating these aspects, we aim to clarify the current performance boundaries of AI-based restoration detection and identify pathways for improving future clinical applicability. Given the dataset size and multi-class complexity, the model is positioned as decision-supportive rather than a standalone diagnostic tool.

**The study’s main contributions to science:**

- A unified benchmark that compares ML, DL, and a hybrid (HGWO-PSO + SVM) pipeline for five-class restoration detection on panoramic radiographs.
- Demonstration that GLCM-based handcrafted texture features, when selected via HGWO-PSO and classified with SVM, yield more balanced and interpretable performance than standalone DL under limited, single-center data conditions.
- An evaluation design focused on generalizability, employing patient-level 80/20 splits, 5-fold cross-validation, and high inter-observer agreement (Cohen’s κ).
- A clinically oriented framing that positions the system as decision-supportive, explicitly considering multi-restoration images and class imbalance, and outlining clear directions for future clinical translation.

The aim of this study is to evaluate and compare multiple AI methodologies, including traditional ML, DL, and a novel hybrid optimization approach (HGWO-PSO + SVM)—for the automatic detection and classification of dental restorations on panoramic radiographs. To the best of our knowledge, this is the first study to integrate HGWO-PSO feature selection within a multi-stage diagnostic workflow for dental imaging. Given the dataset size and multi-class complexity, the model is positioned as decision-supportive rather than a standalone diagnostic tool.

## 2. Materials and Methods

**Figure 1:**
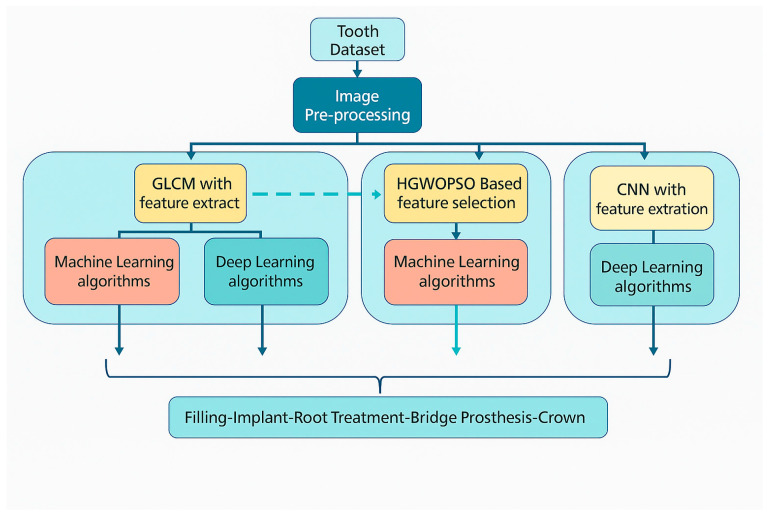
Workflow diagram of the proposed AI system.

The general framework of this work is the development of artificial intelligence methods to automatically detect dental restorations in panoramic radiographs and to classify a range of different and unique dental data. The proposed workflow, shown in Figure 1, is designed as a modular guide that can be easily applied to similar diagnostic studies in dentistry and other medical domains.

The scientific contributions of the proposed methodology can be summarized as follows:

- The dataset used in this study consists of panoramic radiographs obtained from the same imaging center and from the same panoramic X-ray device to ensure standardized imaging quality, since image characteristics may vary across manufacturers and acquisition settings.
- Image enhancement was performed using Histogram Equalization followed by Contrast-Limited Adaptive Histogram Equalization (CLAHE). The combined use of these techniques was empirically chosen to maximize local contrast without losing global brightness balance. Care was taken to avoid over-processing or introducing artifacts; enhancement parameters (clip limit = 2.0, grid size = 8 × 8) were optimized through pilot visual assessment by two dental experts to preserve diagnostic structures.
- A three-stage diagnostic system was implemented, which has not previously been applied to dental datasets:
  1. Feature extraction and classification stage using Gray-Level Co-Occurrence Matrix (GLCM) features combined with conventional ML and DL algorithms.
  2. Hybrid optimization and classification stage using a combined Hybrid Grey Wolf–Particle Swarm Optimization (HGWO-PSO) feature selector and Support Vector Machine (SVM) classifier.
  3. End-to-end deep learning stage using a Convolutional Neural Network (CNN) trained directly on raw images to establish a baseline for comparison.
- The proposed multi-stage system enables evaluation of how handcrafted features, optimization, and learned features interact to affect classification performance.

### 2.1. Dataset and Image Pre-Processing

**Figure 2:**
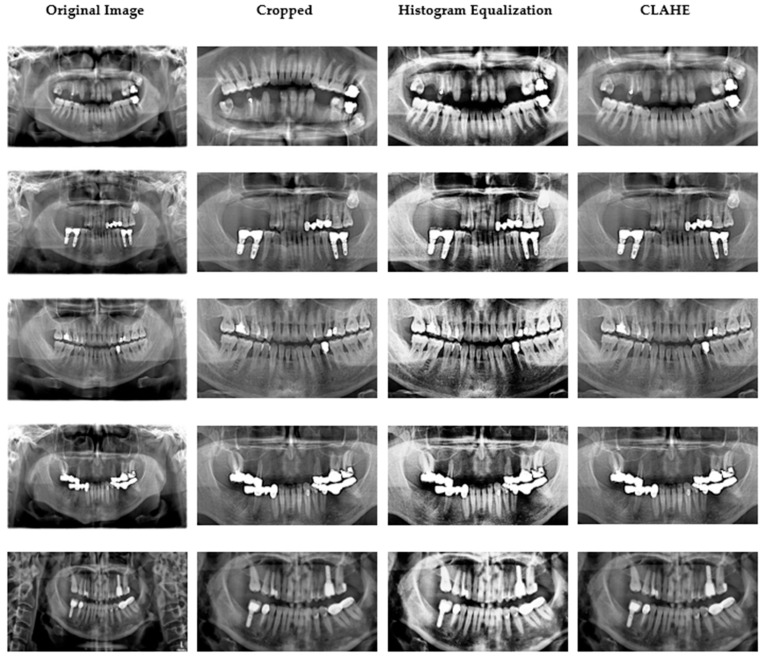
Data samples obtained after image preprocessing.

Ethical approval was granted by the Çankırı Karatekin University Ethics Committee (Meeting date: 10 Nov 2023; Meeting no.: 10; Approval ID: 15f61336b5104878). A total of 353 anonymized panoramic radiographs containing 2137 restorations were retrospectively selected from the archives of the Dental Imaging Center in Ankara. All images were acquired using the Orthopantomograph MORITA Veraviewepocs X-550 under identical exposure parameters (70 kVp, 10 mA, 10 s) [21]. The dataset was divided into 80% training and 20% testing on a per-patient basis to prevent leakage across subjects. Each image was independently annotated by two calibrated dentists (one oral radiologist and one prosthodontist). Inter-observer agreement was quantified on 50 randomly chosen cases with a Cohen’s κ = 0.91, indicating excellent reliability [22]. Five restoration categories were defined: filling, implant, root-canal treatment, fixed partial denture (bridge), and crown. Original images (2775 × 1504 pixels) were cropped to 1700 × 880 pixels to exclude non-diagnostic margins. Subsequently, mild global adjustments of contrast, brightness, and color balance were made using the ImageEnhance module of the Python Pillow library. Thereafter, histogram equalization and CLAHE were applied to accentuate differences among restorative materials and surrounding bone structures [23,24]. CLAHE parameters were carefully tuned (clip limit = 2.0, grid size = 8 × 8) to avoid over-processing artifacts [25]. Representative examples of the original image cropped view, and enhanced outputs (histogram equalization and CLAHE) are shown in Figure 2. In pilot ablations on the training folds, applying histogram equalization (HE) followed by CLAHE produced slightly higher validation stability (macro-F1 ≈ +1.5%) compared with either method alone, while preventing over-enhancement. The additional “Pillow smoothing” step was retained only for visual consistency; detailed quantitative ablations will be released with the code for full reproducibility.

All images were fully anonymized prior to analysis. Patient identifiers (name, ID number, date of birth, acquisition date) were automatically removed at the time of data export by the imaging center. The anonymized dataset was shared under a formal data-use agreement between the Dental Imaging Center and the investigators, in accordance with the approval of the Çankırı Karatekin University Ethics Committee (Approval ID: 15f61336b5104878). All procedures followed the principles of the Helsinki Declaration and GDPR guidelines for data protection and patient privacy.

Each panoramic radiograph may include several restoration types simultaneously. To maintain a consistent training protocol and reduce label sparsity, we assigned a single dominant restoration label per image. Expert annotations (*n* = 2137 restorations) were retained for later error auditing but were not used to generate region-of-interest (ROI) crops or multi-label training sets in this version.

Although expert-level annotations were available for 2137 restorations, these region-level markings were used only for verification and error auditing. Feature extraction in the current study was conducted on the entire radiograph to maintain a uniform processing pipeline across samples. This global-feature approach simplifies the workflow but inherently introduces background noise, as non-relevant anatomical regions contribute to the GLCM and CNN descriptors. In future work, we aim to incorporate these expert annotations for ROI-based or segmentation-driven feature extraction to reduce such noise and improve localization accuracy.

### 2.2. Feature Extraction

Texture features were extracted from the pre-processed grayscale images using the Gray-Level Co-Occurrence Matrix (GLCM) approach [26,27]. Each image was normalized to the intensity range [0, 1], and four standard statistical descriptors—contrast, correlation, energy, and homogeneity—were computed for four angular directions (0°, 45°, 90°, 135°) and averaged to obtain rotation-invariant measures. The resulting feature vectors were standardized by z-score normalization before being supplied to the classifiers. Grayscale images were quantized into 64 gray levels after z-score normalization.

Gray-Level Co-occurrence Matrices (GLCMs) were computed with a pixel offset distance d = 1 at four angles (0°, 45°, 90°, 135°) and symmetrized to ensure rotation invariance. The effectiveness of GLCM-based texture descriptors has been demonstrated in a wide range of imaging domains, including liquid crystal analysis, medical X-ray imaging, histopathology, and ultrasound [21,22,25,28].

Global (whole-image) GLCMs were used instead of sliding-window GLCMs to maintain computational efficiency and consistency across images.

**Figure 3:**
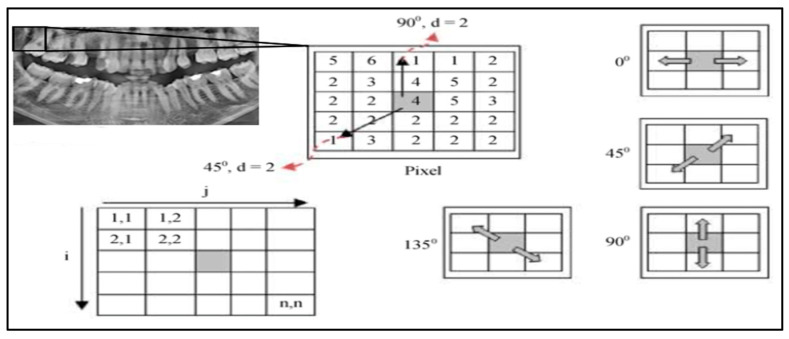
GLCM Illustration with angles of 0°, 45°, 90°, and 135° [25].

From each matrix we extracted five standard texture features—contrast, correlation, energy (ASM), homogeneity, and entropy—and averaged them over all angles to obtain the final descriptor vector. This ensured comparability across images and reduced illumination-related bias (Figure 3).

### 2.3. Hybrid Grey Wolf Optimization-Particle Swarm Optimization (Hgwo-Pso)

In the second stage, the Hybrid Grey Wolf Optimization–Particle Swarm Optimization (HGWO-PSO) algorithm was employed to perform feature selection among the texture descriptors derived from GLCM. The hybridization combines the social hierarchy and adaptive leadership mechanism of the Grey Wolf Optimizer (GWO) with the velocity–position update principle of Particle Swarm Optimization (PSO). The hybrid version was implemented in Python 3.10 using NumPy and SciPy libraries [29,30,31].

Each wolf represents a potential subset of texture features. The position vector Xᵢ = (xᵢ_1_, xᵢ_2_, …, xᵢd) corresponds to a binary inclusion mask, where “1” indicates the selected feature. Fitness was evaluated based on classification accuracy (Acc) and feature-reduction ratio (Fr) according to Equation (1):

F = 0.9 × Acc + 0.1 × (1 − Fr) (1)

A population of 10 wolves and 100 iterations yielded the most balanced performance during pilot runs. Increasing either parameter led to longer computation times and a tendency toward overfitting, whereas smaller populations degraded exploration ability.

The inertia weight w was linearly decreased from 0.8 → 0.4, and the learning coefficients were set to c_1_ = c_2_ = 2, following common benchmark settings [32]. The HGWO-PSO algorithm dynamically adjusts the top three wolves (α, β, δ) and updates the remaining agents according to Equation (2):

X (t + 1) = wV (t) + c1r1 (Pbest − X (t)) + c2r2 (Gbest − X (t)) (2)

where r_1_ and r_2_ are random vectors in [0, 1]. This hybrid update enhances both global search (GWO behavior) and local exploitation (PSO velocity). After convergence, the optimal feature subset was used as input to the Support Vector Machine (SVM) classifier with a radial basis function (RBF) kernel. An example of the GLCM feature subset selected by the HGWO-PSO algorithm for SVM classification is presented in Table 1.To prevent information leakage and ensure fair evaluation, the HGWO-PSO feature selection procedure and SVM hyperparameter tuning were both performed within each training fold of the cross-validation loop. Specifically, for every fold, the feature-selection algorithm was run using only the training portion of that fold to identify the optimal subset of features and corresponding SVM parameters (C and γ). The held-out validation and final test sets were never included in any stage of optimization or feature selection, ensuring complete data isolation between training and evaluation phases.A grid-search procedure tuned the penalty parameter C and kernel width γ. The optimized SVM achieved an average 6.2% accuracy improvement while reducing the feature dimension by 45%.

### 2.4. Classification with Machine Learning and Deep Learning

**Figure 4:**
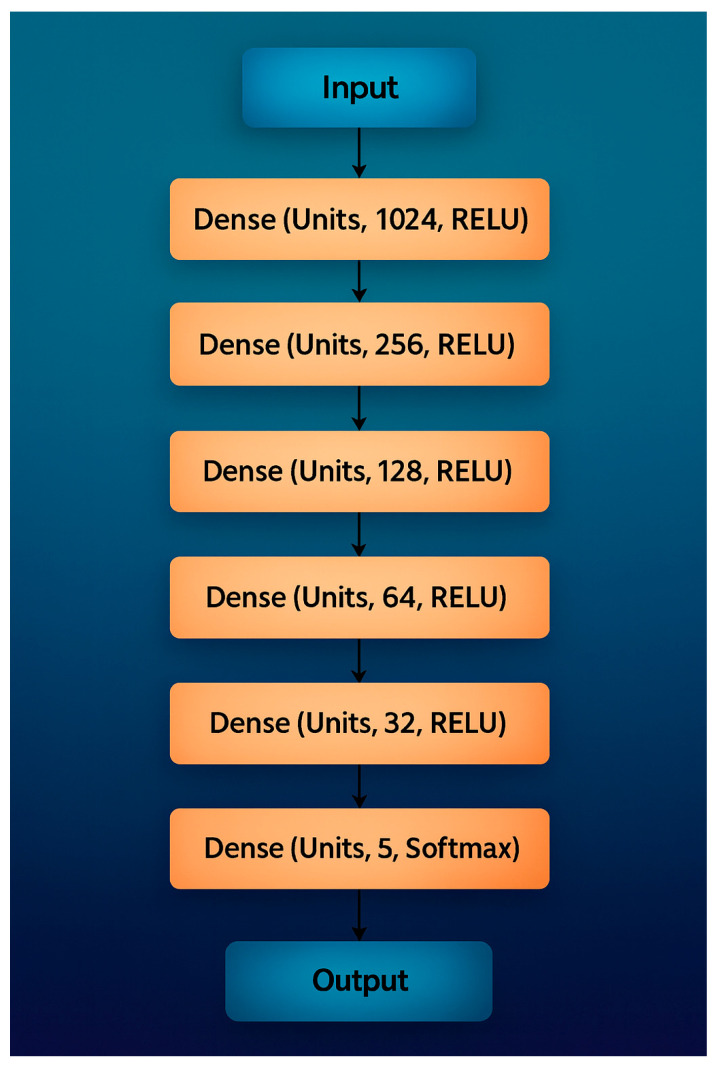
Recommended CNN Architecture for classification.

This section compares the performance of classical machine-learning (ML) models and deep-learning (DL) methods for classification. Five ML algorithms were tested: K-Nearest Neighbors (K-NN), Support Vector Machine (SVM), Decision Tree (DT), Random Forest (RF), and the hybrid HGWO-PSO + SVM model, followed by a Convolutional Neural Network (CNN) baseline (Figure 4, Table 2).

The CNN baseline was implemented in TensorFlow 2.12 (CUDA 11.8/cuDNN 8.9) and trained with Adam (learning rate 1 × 10^−4^), batch size 32, for up to 50 epochs with early stopping (patience 5); data augmentation included horizontal flips, ±10° rotations, and ±15% brightness variation.

The K-NN algorithm is a distance-based classifier widely used for pattern recognition due to its simplicity and interpretability. The optimal k parameter was empirically determined via grid search in the range k = 3–11, with k = 5 yielding the highest validation accuracy [32,33].

The SVM algorithm [34] employs a convex optimization framework to identify a hyperplane that maximizes inter-class margins, thereby minimizing structural risk. For this work, an RBF kernel was selected; hyperparameters C = 10 and γ = 0.1 were optimized by cross-validation.

Decision Tree (DT) and Random Forest (RF) classifiers were built using scikit-learn with Gini impurity and a maximum depth of 20 [35,36,37,38]. The RF ensemble consisted of 100 estimators trained on bootstrapped samples, improving robustness to overfitting.

CNNs are state-of-the-art DL models for image classification [39,40,41]. The proposed CNN (Figure 4) follows a sequential design with increasing filter depth to extract both low-level and high-level features. After the input image was resized to 224 × 224 × 3, convolutional layers with filter counts 32, 64, 128, 256, 512 were applied. Each block used ReLU activation and 2 × 2 max-pooling. To reduce overfitting, dropout layers (0.2 and 0.1) were inserted after the second and final dense blocks. Fully connected layers of 256 and 512 neurons preceded the SoftMax output of 5 classes (filling, implant, root-canal treatment, bridge, crown).

All experiments were implemented in TensorFlow 2.12 with CUDA 11.8/cuDNN 8.9 on an NVIDIA RTX-A4000 GPU (16 GB) under Ubuntu 22.04. Model training used Adam optimizer (learning rate = 1 × 10^−4^), batch size = 32, 50 epochs, and early stopping (patience = 5). Data augmentation included random horizontal flips, ±10° rotations, and ±15% brightness variation. Table 2 summarizes the proposed CNN architecture, showing the main layer groupings, kernel sizes, strides, activations, and dropout ratios. This tabular format improves clarity while preserving all architectural details.

### 2.5. Performance Measurements

To evaluate the classification models, both global and per-class performance metrics were computed: Accuracy, Precision, Recall, and F1-score, together with confusion matrices [39,40,41]. These metrics allow comparative interpretation of model reliability and inter-class discrimination. Given the inherent class imbalance among restoration categories, relying solely on overall accuracy could obscure clinically relevant misclassifications. Therefore, macro-averaged precision, recall, and F1-scores were used to ensure balanced evaluation across all classes. In addition, confusion matrices were analyzed to highlight errors with differing clinical consequences. For instance, misclassifying a crown as a bridge is clinically less critical than missing an implant, which may lead to more significant diagnostic oversight. This multi-metric approach provides both statistical and clinical interpretability of model behavior, ensuring that performance evaluation aligns with real-world dental diagnostic priorities. To mitigate class imbalance, class-weight correction was applied and macro-averaged Precision, Recall, and F1-scores were reported alongside Accuracy. Accuracy was computed as (TP + TN)/(TP + TN + FP + FN) (Equation (3)).

Accuracy = (TP + TN)/(TP + TN + FP + FN) (3)

In addition, the per-class metrics were defined as (Equations (4)–(6)).

Precision = TP/(TP + FP) (4)

Recall = TP/(TP + FN) (5)

F1-score = 2 × (Precision × Recall)/(Precision + Recall) (6)

**Figure 5:**
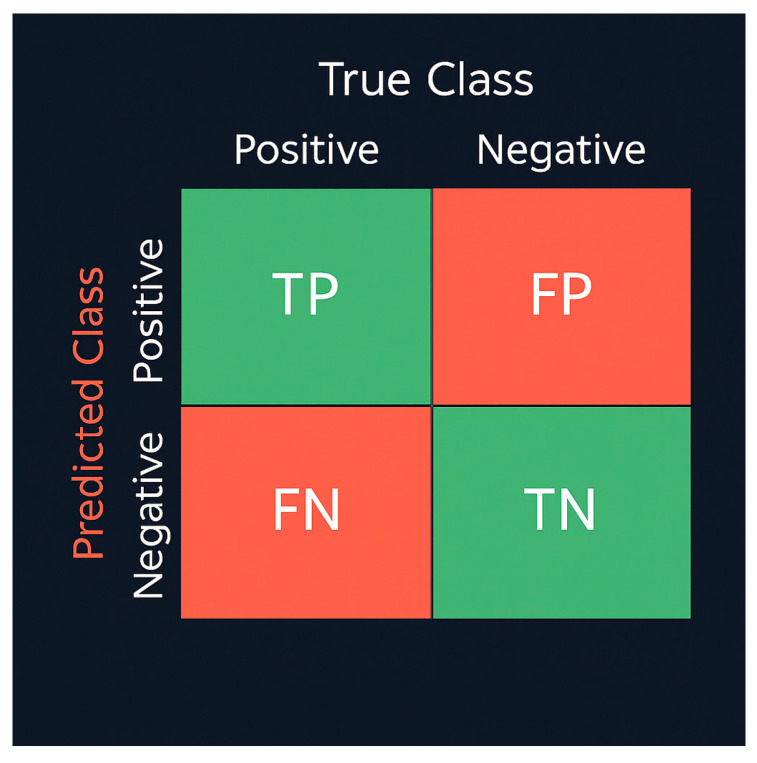
Confusion Matrix Calculation Table.

Here, TP, TN, FP, and FN denote the counts of true positives, true negatives, false positives, and false negatives, respectively. To provide a clearer visual understanding, the confusion-matrix scheme is illustrated in Figure 5, which conceptually summarizes prediction outcomes for all classes.

A 5-fold cross-validation was conducted on the training subset (80% of data) to verify model stability. All reported metrics represent the mean performance across the five cross-validation folds, each trained on distinct patient partitions. To estimate the variability of the results, we computed the standard deviation and 95% confidence intervals by stratified bootstrap resampling (1000 iterations) on the aggregated test predictions of each model.

This approach provides an unbiased measure of statistical uncertainty across folds. Additionally, a post hoc power analysis (G*Power 3.1, α = 0.05, β = 0.2) verified sufficient sample size (*n* = 353) with power > 0.84. All computations and visualizations were performed in Python 3.10 using NumPy 1.26, scikit-learn 1.3, matplotlib 3.8, and seaborn 0.13. In terms of computational efficiency, the hybrid HGWO-PSO + SVM pipeline required approximately 1.6 s per image for feature extraction and classification, while the CNN baseline inference time was 0.9 s per image on an NVIDIA RTX A4000 GPU. The HGWO-PSO phase converged within 2.5 min on average for 100 iterations. Although the CNN required higher memory usage (~2.3 GB) due to backpropagation, both methods achieved clinically acceptable runtime performance for real-time decision-support applications.

## 3. Results

In this section, the performance of all machine-learning and deep-learning models is presented. Table 3 lists the GLCM features selected by the HGWO-PSO procedure, and Table 4 reports the quantitative classification results (Precision, Recall, F1-score, Accuracy) for K-NN, SVM, Decision Tree (DT), Random Forest (RF), the hybrid HGWO-PSO + SVM, and the CNN baseline trained on panoramic radiographs.

The HGWO-PSO + SVM model achieved the highest overall accuracy (73.15%), outperforming all other models (see Table 4). CNN reached 68.52%, while K-NN obtained the lowest performance (53.70%). The difference between HGWO-PSO + SVM and CNN was statistically significant (paired *t*-test, *p* < 0.05). This indicates that the hybrid optimization method improved discriminative feature selection, reducing redundancy and noise in the GLCM descriptors [42,43].

**Figure 6:**
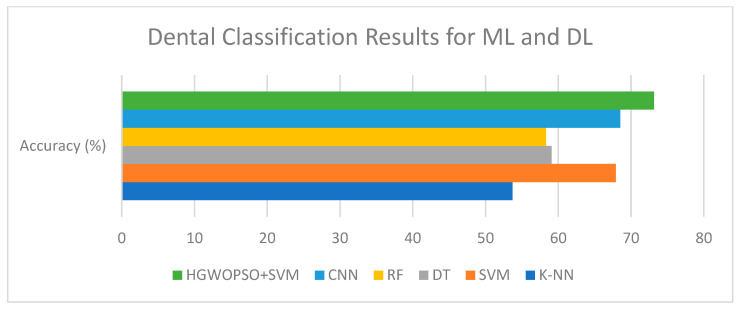
Comparison of classification performances of AI algorithms.

Figure 6 illustrates the classification accuracy of the evaluated machine-learning and deep-learning models. Among all methods, the hybrid HGWO-PSO + SVM achieved the highest accuracy (73.15%), followed by CNN (68.52%) and SVM (67.89%). The reported results correspond to the average of five cross-validation folds on the training set, with independent per-patient test partitions. Across folds, the hybrid HGWO-PSO + SVM model showed a standard deviation of ±2.1% in accuracy. Bootstrap resampling (1000 iterations) of the test predictions yielded 95% confidence intervals of [71.1%, 75.2%] for accuracy and comparable ranges for macro-F1. These statistics confirm that the observed improvements are consistent rather than partition-specific. Traditional classifiers such as DT (59.09%), RF (58.33%), and K-NN (53.70%) showed relatively lower performance, demonstrating the advantage of the optimization-based hybrid approach over both conventional ML algorithms and the DL baseline.

**Figure 7:**
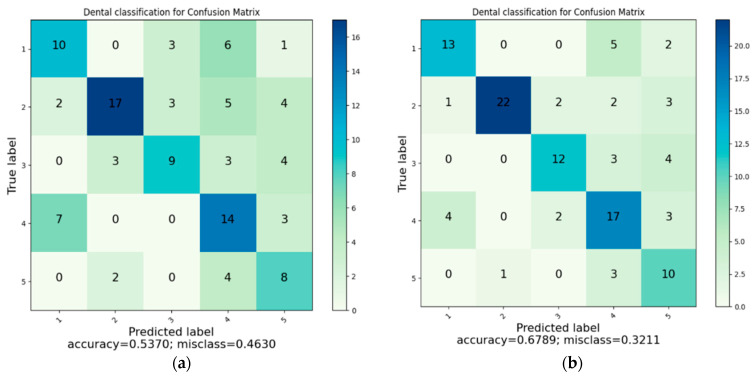

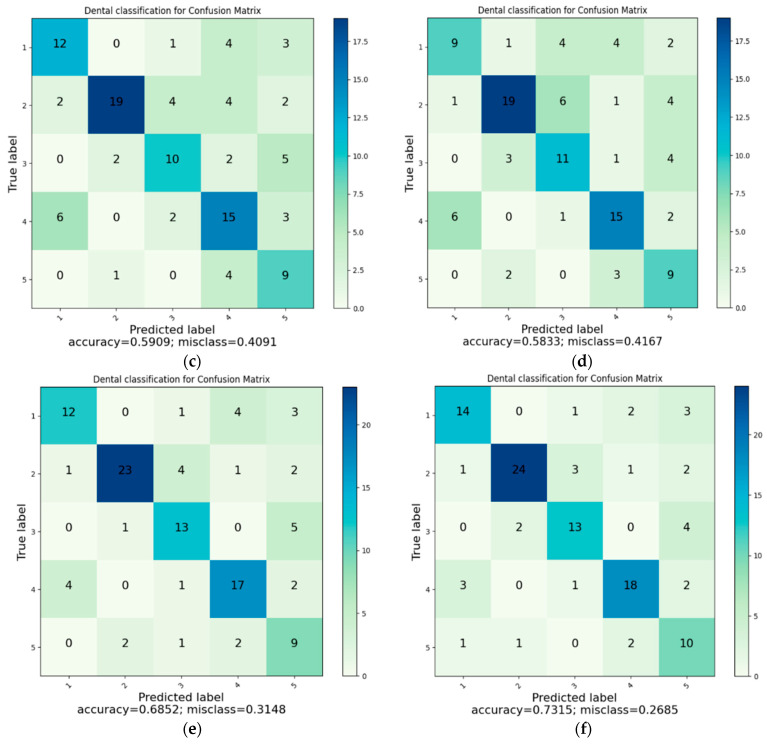
(**a**) Classification with K-NN. (**b**) Classification with SVM. (**c**) Classification with DT. (**d**) Classification with RF. (**e**) Classification with CNN. (**f**) Classification with HGWOPSO + SVM.

Figure 7 presents the composite confusion matrix summarizing the classification performance of all evaluated models. Accuracy was computed as (TP + TN)/(TP + TN + FP + FN) (Equation (1)). Compared with other models, K-NN exhibited the highest misclassification between crown and filling classes, resulting in the lowest overall precision. In contrast, the proposed HGWO-PSO + SVM model showed the most balanced performance across all categories. To enhance visual clarity and reduce redundancy, the six separate confusion matrices were consolidated into a single figure. 

The confusion-matrix tables indicate that the margin of error is greater in dental filling, bridge prosthesis, and crown classes. In addition, because more than one restoration may appear in a single image, the methods can have difficulty distinguishing restorations for classification. Despite these challenges, the CNN and HGWO-PSO + SVM methods predicted all classes with acceptable misclassification levels.

Notably, metallic restorations such as large amalgam fillings, full-metal crowns, and orthodontic appliances occasionally produced bright artifacts and beam-hardening effects on panoramic radiographs. These high-density regions sometimes resulted in false-positive predictions (e.g., misclassifying a metallic filling as a crown) or false-negative detections due to signal saturation. Such artifacts reduce local texture contrast, which may limit the discriminative power of both handcrafted and CNN-based features. Future work will include artifact-aware preprocessing and region-based modeling to mitigate this effect.

## 4. Discussion

This section interprets the comparative results and their clinical implications rather than repeating numerical findings presented in the Results. Overall, the hybrid HGWO-PSO + SVM model showed consistent improvement across metrics, confirming that optimization-assisted feature selection improves diagnostic reliability. It therefore highlights the methodological contribution and decision-support relevance of the proposed approach, particularly for limited-data clinical imaging scenarios.

According to the present study, the highest classification accuracy (73.15%) was achieved when HGWO-PSO was combined with SVM; the CNN achieved 68.52%; SVM 67.89%, DT 59.09%, RF 58.33%, and K-NN 53.70%. The superior performance of HGWO-PSO + SVM over CNN can be attributed to the optimized feature-selection process: by reducing redundant and noisy features, HGWO-PSO enhanced the discriminative capacity of the feature set, enabling SVM to establish clearer decision boundaries between restoration classes. In contrast, CNN learned directly from raw image data, which—given the limited dataset size—may incorporate irrelevant patterns. Consequently, HGWO-PSO + SVM provided robust, noise-resistant classification.

The algorithmic workflow developed here includes a multi-stage evaluation using both ML and DL techniques. In the first stage, basic classification is performed with handcrafted texture features; in the second, HGWO-PSO performs feature selection before final classification with SVM, yielding higher accuracy even for visually similar, hard-to-distinguish categories. Prior studies have reported strong CNN performance for crowns/bridges detection [41,42]. Our results refine this picture by showing that optimization-assisted feature selection can further enhance performance on limited, single-center datasets.

Researchers have used various AI-based programs to detect and classify dental restorations effectively. Rubiu et al. reported 98.4% success using Mask-RCNN on 1000 panoramic radiographs [43]. Another study using a Matlab^®^-based algorithm on 83 panoramics achieved identification accuracies ranging from 83.1% to 100%, with 100% for crowns and 99.9% for implants [12]. Bonfanti-Gris et al. analyzed 300 panoramics using online software and reported 100% for crowns and 99.9% for implants [44]. Altan et al. examined 5126 panoramics, yielding sensitivities of 74% for crowns and 84% for bridges; lower sensitivity was attributed to dataset scale and image-quality variability [45]. A separate YOLO-v5x study reported high sensitivity for tooth and implant-supported crowns, pontics, and implants, indicating strong potential for object detection applications in dentistry [46].

This paper offers a comprehensive evaluation of six ML/DL algorithms for detecting dental restorations, prostheses, and implants. Unlike prior studies focusing on a single method, we compare all models under a unified protocol. As illustrated in Figure 7, error rates were higher for fillings, bridge prostheses, and crowns, and images containing multiple restorations further complicated classification. Even so, CNN and HGWO-PSO + SVM achieved acceptable misclassification across all classes, supporting their role as decision-support tools in clinical practice. Clinically, most confusion occurs between radiopaque restorations (e.g., crowns vs. bridges), which typically do not change treatment plans but affect descriptive reporting. From a clinical perspective, not all classification errors have equal relevance. For instance, misclassifying a crown as a bridge has minimal diagnostic impact, whereas missing an implant could lead to clinically significant oversights. In this context, the use of macro-averaged precision and recall metrics allowed us to highlight these clinically important discrepancies and ensure balanced model assessment across all restoration types. Therefore, the proposed system should be regarded as a diagnostic decision-support tool assisting clinicians rather than an autonomous diagnostic solution.

A limitation of this study is the relatively small dataset (353 panoramics; 2137 restorations), which may restrict generalizability. DL approaches typically require larger and more diverse datasets to capture variability in anatomy and imaging conditions; thus, reported accuracies should be interpreted with caution regarding broader populations. Future research will expand to multi-center, multi-device datasets to enhance robustness and external validity and will explore generalization to other modalities (intraoral radiographs, CBCT). The dataset split was 80/20 per-patient to prevent subject-level leakage.

An additional limitation is that all images were obtained from a single imaging device (MORITA Veraviewepocs X-550) at one clinical center. While this ensured standardized acquisition parameters, it may limit the generalizability of the model to other radiographic systems and devices with different dynamic ranges or exposure characteristics. A multi-center, multi-device validation would therefore be essential to evaluate clinical scalability and robustness across different imaging environments.

Another limitation concerns task formulation. Although each image may contain multiple restorations, we modeled the task as single-label, image-level classification and computed global features over the entire image. This approach simplifies training but introduces label noise and overlooks spatial localization. Future work will reformulate the problem as multi-label classification and employ ROI- or segmentation-based networks to capture multiple coexisting restorations within the same radiograph. Additionally, the present analysis used global image-level features rather than localized regions of interest (ROIs). Although region annotations were available, they were not used for feature computation. This design simplifies implementation but allows non-diagnostic areas—such as bone background or soft-tissue shadows—to influence the extracted texture statistics. Employing ROI-specific or patch-based feature extraction in future versions could substantially reduce background bias and increase discriminative power for overlapping restorations.

Although the HGWO-PSO + SVM approach achieved 73.15% accuracy—moderate relative to some studies reporting >80–90%—the hybrid model consistently outperformed conventional ML algorithms and CNN on our data, as optimization-based selection reduced noise and improved feature discriminability. Regarding the deep-learning baseline, it should be noted that training a CNN model from scratch on 353 panoramic images represents a relatively small-data scenario, which naturally limits generalization capacity. Consequently, the baseline CNN may exhibit underfitting or overfitting tendencies compared with transfer learning or larger-scale pretrained architectures. The observed superiority of the hybrid HGWO-PSO + SVM model therefore reflects its robustness in limited-data conditions rather than an absolute advantage over optimized deep-learning frameworks. In future work, we plan to incorporate pretrained CNNs (e.g., ResNet-50, EfficientNet) and transformer-based backbones (e.g., ViT, Swin-Transformer) to provide stronger and fairer baseline comparisons under identical experimental settings. Clinically, these findings suggest that optimization-assisted models can reduce dentist workload, standardize interpretation, and accelerate treatment planning. Looking ahead, practical applications include integrating HGWO-PSO + SVM into computer-aided diagnosis systems and extending to CBCT or intraoral radiographs—reinforcing its potential for routine clinical dentistry—while maintaining a decision-supportive rather than standalone stance. Furthermore, this study utilized data collected from a single imaging center using a uniform panoramic device. While this ensured standardized image quality and consistent acquisition parameters, it also limits the generalizability of the results to other imaging systems or clinical environments. Device-specific differences in exposure, detector sensitivity, or post-processing algorithms could influence model performance. To enhance robustness and external validity, future research will include multi-center datasets, cross-device experiments, and domain adaptation analyses to verify model transferability across diverse clinical settings.

In addition, the current study did not include external validation or transfer learning baselines. This was primarily due to the absence of publicly available, multi-center panoramic datasets with comparable labeling schemes. Future work will therefore incorporate transfer learning with pretrained CNN backbones (e.g., ResNet-50, EfficientNet) and transformer architectures (e.g., Vision Transformer, Swin-Transformer) as external baselines to assess generalization under identical experimental conditions. These extensions will enable fairer comparisons and provide stronger evidence of clinical robustness across institutions.

Recently, transformer-based and segmentation-driven architectures such as Vision Transformer (ViT), Swin-Transformer, and Swin-UNet have achieved remarkable results in medical image analysis, particularly for pixel-level tasks. While our current work focuses on handcrafted and global texture features, these transformer-based models can provide finer spatial attention and region-based understanding. In this context, our hybrid optimization framework can serve as a complementary lightweight alternative for small-scale datasets, whereas transformer baselines will be integrated in future experiments for a more direct comparison.

## 5. Conclusions

The AI framework combining deep-learning and machine-learning components demonstrated strong performance in detecting and classifying dental restorations. Integrating HGWO-PSO with SVM improved feature selection and achieved the highest accuracy among evaluated models, supporting the use of AI for radiological diagnosis and treatment planning in restorative dentistry. Accordingly, the model is positioned as an assistive decision-support framework to complement, not replace, expert radiological judgment.

These techniques have the potential to enhance efficiency, improve diagnostic consistency, and ultimately contribute to better patient care. Nevertheless, additional work is required to raise performance, especially under class imbalance and multi-restoration images—and to assess applicability across different panoramic imaging systems and clinical settings. Given the dataset size and multi-class complexity, the system should be regarded as decision-supportive rather than a stand-alone diagnostic tool.

**Future work:** To improve performance and clinical generalizability, future studies will (i) expand the dataset to multi-center and multi-device collections to evaluate robustness across different imaging systems, acquisition parameters, and patient populations; (ii) incorporate segmentation-based architectures such as U-Net and Swin-UNet to localize multiple restorations within each panoramic radiograph and reduce background interference; (iii) evaluate transformer-based baselines including Vision Transformer (ViT) and Swin-Transformer for comparative validation with the proposed hybrid HGWO-PSO + SVM framework; and (iv) assess computational efficiency (runtime, FLOPs, memory footprint) and prospective clinical integration within digital-dentistry workflows through user-interface prototypes and clinician feedback.

The proposed AI system shows clear potential for clinical integration: automating restoration detection could reduce workload, standardize workflows, and minimize inter-observer variability. These benefits may allow clinicians to focus more on treatment planning and patient care while facilitating the management of large radiographic datasets in clinical and research settings.

## Figures and Tables

**Table 1:** Example of the GLCM feature subset selected by HGWO-PSO for SVM classification.

Feature	Description	Selected (1/0)	Weight
Contrast (0°)	Local gray-level variance	1	0.82
Correlation (90°)	Pixel dependency	1	0.77
Energy (135°)	Uniformity measure	0	0.15
Homogeneity (45°)	Local similarity	1	0.64
Entropy	Texture randomness	1	0.73

**Table 2:** Compact representation of the proposed CNN architecture.

Stage	Layers Included	Kernel Size(s)	Stride	Activation	Dropout
Convolution Block 1	Conv (32 filters) + Max Pooling	3 × 3, 2 × 2	2 January	ReLU	–
Convolution Block 2	Conv (64 filters) + Max Pooling	3 × 3, 2 × 2	2 January	ReLU	–
Convolution Block 3	Conv (128 filters) + Max Pooling	3 × 3, 2 × 2	2 January	ReLU	–
Convolution Block 4	Conv (256 filters) + Conv (512 filters) + Pooling	3 × 3, 3 × 3, 2 × 2	1 January 2002	ReLU	–
Fully Connected	Dense	–	–	ReLU	0.2/0.1
Output	Dense (5 classes)	–	–	SoftMax	–

**Table 3:** Features selected with HGWO-PSO.

Feature	Number
Correlation135	9
Homogeneity	10
Homogeneity45	11
Homogeneity90	12
ASM	21

**Table 4:** Comparison of classification results.

Model	Precision	Recall	F1-Score	Accuracy (%)
K-NN	0.533	0.533	0.533	53.70
SVM	0.690	0.690	0.690	67.89
DT	0.596	0.596	0.596	59.09
RF	0.575	0.575	0.575	58.33
CNN	0.675	0.675	0.675	68.52
HGWO-PSO + SVM	0.728	0.728	0.728	73.15

## Data Availability

The dataset used in this study contains patient-derived panoramic radiographs and is therefore subject to ethical and privacy restrictions under the approval of the Çankırı Karatekin University Ethics Committee (Approval ID: 15f61336b5104878). De-identified image samples and all code scripts used for data preprocessing, feature extraction, model training, and statistical analysis are available from the corresponding author upon reasonable request for academic research purposes. Additionally, the full implementation of the HGWO-PSO + SVM and CNN baselines will be publicly released via an open access GitHub repository following acceptance of this manuscript, ensuring full reproducibility of the reported experiments. All analyses were performed using Python 3.10, NumPy 1.26, SciPy 1.11, scikit-learn 1.3, TensorFlow 2.12 (CUDA 11.8/cuDNN 8.9), and Matplotlib 3.8.

## Abbreviations

The following abbreviations are used in this manuscript:

Mask-RCNN	Mask Region-Based Convolutional Neural Network
FN	False Negative
FP	False Positive
TN	True Negative
TP	True Positive
DT	Decision Tree
ASM	Angular Second Moment
CLAHE	Contrast-Limited Adaptive Histogram Equalization
ANN	Artificial Neural Network
K-NN	k-Nearest Neighbors
CNN	Convolutional Neural Network
SVM	Support Vector Machine
HGWO-PSO	Hybrid Grey Wolf–Particle Swarm Optimization
DL	Deep Learning
ML	Machine Learning
AI	Artificial Intelligence
